# Bioactive Steroids and Saponins of the Genus *Trillium*

**DOI:** 10.3390/molecules22122156

**Published:** 2017-12-05

**Authors:** Shafiq Ur Rahman, Muhammad Ismail, Muhammad Khurram, Irfan Ullah, Fazle Rabbi, Marcello Iriti

**Affiliations:** 1 Department of Pharmacy, Shaheed Benazir Bhutto University, Sheringal, Dir 18000, Pakistan; drmkhurram@sbbu.edu.pk; 2 Department of Pharmacy, University of Peshawar, Peshawar 25120, Pakistan; m_ismail@upesh.edu.pk (M.I.); fazlerabbi_rph@uop.edu.pk (F.R.); 3 Department of Pharmacy, Sarhad University of Science and Information Technology, Peshawar 25120, Pakistan; irfanu3@gmail.com; 4 Department of Agricultural and Environmental Sciences, Milan State University, 20133 Milan, Italy

**Keywords:** bioactive phytochemicals, cytotoxic activity, anti-inflammatory activity, analgesic activity, antifungal activity

## Abstract

The species of the genus *Trillium* (Melanthiaceae alt. Trilliaceae) include perennial herbs with characteristic rhizomes mainly distributed in Asia and North America. Steroids and saponins are the main classes of phytochemicals present in these plants. This review summarizes and discusses the current knowledge on their chemistry, as well as the in vitro and in vivo studies carried out on the extracts, fractions and isolated pure compounds from the different species belonging to this genus, focusing on core biological properties, i.e., cytotoxic, antifungal and anti-inflammatory activities.

## 1. Introduction

Natural products obtained from plants have played remarkable role in drug discovery and improvement of health care system [1,2,3,4,5,6]. According to the World Health Organization (WHO) estimate, about 80% of world population relies on natural sources for their primary health care need while the remaining 20% of the population uses integrated natural sources [7]. Even at the dawn of 21st century, 11% of the 252 drugs considered as basic and essential by the WHO were exclusively of flowering plants origin [8]. At present, the area of cancer and infectious diseases are mostly dependent on natural products, and among the 175 approved anti-cancer drug molecules, 85 (49%) are either natural products or their derivatives [3].

In scientific literature around the world, more than 35,000 plant species have been reported to be used in different human cultures for medicinal purposes [9]. Nevertheless, this number could be much higher, as knowledge of indigenous use of medicinal plants mainly passes verbally from one generation to another and largely remains undocumented. Among the 250,000 reported higher plant species, only 5–15% has been scrutinized for their bioactive molecules [10]. Therefore, medicinal plants represent an area under focus since their secondary metabolites encompass a significant number of drugs used in current therapeutics and their potential as source of new medicines is beyond any doubts.

Saponins are steroid or triterpene glycosides widely distributed in the plants that possess hemolytic properties and poisonous effects [11]. The aglycone part (sapogenin) of saponins may have steroid or triterpenoid nuclei, based on whom saponins are generally classified. Steroidal saponins are less common and usually found in monocotyledonous plants as compared to triterpenoid saponins, which are extensively distributed and found in dicotyledonous plants [12]. The basic skeleton of steroidal sapogenins (27C) may be either 6-ring spirostane or 5-ring furostane, while triterpenoid sapogenins (30C) are structurally different and often consist of five or rarely four units. In general, at C-3 of aglycone moiety, the glycone (sugar) is attached, including one to several monosaccharide units. The attached sugar chains may be from one to three, either straight, branched or both. The presence of different substituents in the sapogenin as well as the composition, linkage and number of sugar moieties account for structural diversity of saponins [13].

Similarly, the structural complexity of the saponins accounts for their diverse physicochemical, pharmacological and biological properties as well as their commercial relevance as promising molecules with several applications in food, cosmetic, pharmaceutical and health fields [14]. Indeed, saponins have been investigated for the development of new natural medicines and to prove the efficacy of traditional herbal medicines. Crude drugs containing saponins that have less irritating effects following oral administration are generally used as expectorant and antitussive agents [11]. It is worth mentioning that many saponins have been reported to exhibit significant anti-inflammatory, anti-nociceptive, antipyretic, anti-allergic and anti-cancer properties [15,16].

Steroids are a group of secondary metabolites derived from cholesterol, showing diverse chemical structure and biological functions. Almost all steroid molecules possess the same basic perhydroxyl cyclopentenophenanthrene skeleton. The differences in the basic skeleton and the attachment of different groups result in various classes of steroids [17]. Steroids have many pharmacological applications and the research is continuing to find out about these metabolites, as potential lead compounds in drug design and discovery [18]. For instance, ecdysteroids are polyhydroxy steroids produced by certain plants including those belonging to the genus *Trillium*. Plants containing ecdysteroids possess noticeable pharmacological properties as anabolic, antidiabetic, analgesic, anti-inflammatory and anthelmintic activities [18,19].

## 2. The Genus *Trillium*

*Trillium* is the most important genus belonging to the family Melanthiaceae alt. Trilliaceae. Steven Elliott in 1817 wrote: “The family Trilliaceae is an attractive one. A spiral of leaves at the peak of a stem, sustaining solitary flower; it encloses and covers numerous species”. The genus *Trillium* consists of perennial herbs with characteristic rhizomes that are horizontal or erect, semierect, branched or faintly unbranched, compressed to shortened, elongated to bulky and fleshy, distal end pointed or premorse; the apex bears large terminal shoot/bud. Stem has leaf-sheaths and brown scales at the base. Leaves are three located at the top of the main stem. Flowers are totally to partly pedicellate, sessile and syncarpous. The genus comprises about forty-eight interrelated species in eastern North America and temperate areas of Asia, as well as in western North America [20]. Most of the *Trillium* species are related with deciduous forests (Arcto-Tertiary flora), which have continued with remarkable changes in geographical ranges since the early Tertiary period in the northern hemisphere. At present, *Trillium* species are limited to one of three geographical areas: Asia, western North America and eastern North America [21]. Some important species of the genus *Trillium* with specific characteristics [22,23,24] are reported in Table 1.

## 3. Phytochemicals of the Genus *Trillium*

The genus *Trillium* is a rich source of bioactive phytochemicals as steroids, saponin derivatives and flavonoids [25,26,27,28,29]. Thus far, several steroidal saponins have been isolated and purified from the plants belonging to this genus and, at present, novel metabolites/phytochemicals from *Trillium* ssp. are investigating using the latest technologies [30,31,32,33,34]. The secondary metabolites/phytochemicals of species belonging to the genus *Trillium* are reported in Table 2.

## 4. Medicinal Importance and Biological Activities of the Genus *Trillium*

Plant species belonging to the genus *Trillium* have been extensively used as a remedy for various diseases in different traditional healing systems and several preclinical studies have corroborated these uses. The reported biological/pharmacological activities of different species indicate the promising potential of crude extracts, solvent fractions and isolated pure compounds.

The rhizomes of *T. erectum*, named beth roots, have been used in folk medicine for the treatment of hemorrhages from uterus, urinary tract and lungs [43]. *T. tschonoskii* has been traditionally used in China for at least one thousand years [44,45]. Dried rhizomes of this plant species have been used as herbal remedy for treatment of hypertension, neurasthenia, giddiness, headache, removing carbuncles and ameliorating pains [46,47]. The anticancer and pro-apoptotic activities of *n*-BuOH extract against human lung cancer cells have also been demonstrated [45].

The ethanol, ethyl acetate and butanol extracts of *T. tschonoskii* significantly suppressed the edema of rat hind paw swelling elicited by injection of carrageenan [48]. Finally, *T. tschonoskii* improved learning and memory in rats, by enhancing the expression of anti-oxidase enzymes [49].

Ethanol extract from rhizomes and aerial parts of *T. grandiflorum* exhibited antifungal activity [40]. The rhizome of *T. govanianum* is commonly known as “matar zela” or “teen patra” in Pakistan, and “nag chatri” in India [19]. In folk medicine, *T. govanianum* is used to cure dysentery and boils; in wound healing, and menstrual and sexual disorders; and as anti-inflammatory and antiseptic agent [50]. The powdered roots are used as body and sexual tonic [51]. Noteworthy, analgesic, anti-inflammatory, antifungal, free radical scavenging, β-glucuronidase inhibitory activities as well as cytotoxicity against prostate and cervical carcinoma cells of *T. govanianum* have been recently reported [19,29,50].

## 5. Bioactivities of the Genus *Trillium* Phytochemicals

The isolated secondary metabolites of the genus *Trillium* mostly belong to the chemical class of steroidal saponins and steroids, including ecdysteroids, even if flavonoids and trihydroxy fatty acids have also been reported. The isolated compounds exhibited a remarkable potential when tested in different in vitro and in vivo assays. Thus far, among the tested steroidal saponins, both spirostanol and furostanol saponins, many of them exhibited relevant cytotoxicity against different cancer cell lines, few showed high potential against tested fungal strains, while some of them possessed antioxidant and COX-2 inhibitory activity, as reported in Table 3.

In addition, the biological activities of steroidal saponins mainly depend on their aglycone moieties (steroidal sapogenins) as well as the number and structure of monosaccharide units in their sugar chains. A slight structural diversity endorses a significant difference on their antifungal and cytotoxic properties, i.e., spirostanol saponins exhibit a higher antifungal potential in comparison to their analogous furostanol saponins. Therefore, it is worth mentioning that further detailed studies on the structure–activity relationships (SAR) and molecular/biochemical targets of steroidal saponins are required to explore the therapeutic potential of this important class of natural products as leads for new drug discovery.

## 6. Conclusions and Future Perspectives

Thus far, more than 40 steroidal saponins having spirostane and furostane type aglycons from the genus *Trillium* have been isolated. Their structure was determined by the use of spectroscopic techniques, including fast atom bombardment mass spectrometry (FABMS) and extensive 2D nuclear magnetic resonance (NMR) experiments (COSY, TOCSY, NOESY, HSQC, and HMBC). The existing data strongly suggest that plants belonging to the genus *Trillium* are rich source of steroids and saponins and possess a therapeutic potential in the management of cancers, fungal infections, inflammatory and painful disorders. Noteworthy, most of the plant species are still unexplored, and some are under investigation; therefore, further comprehensive studies are needed to screen new sources of phytochemicals to develop promising phytotherapeutics effective in the treatment of chronic degenerative and infectious diseases. Finally, the most promising *Trillium* secondary metabolites have to be investigated in humans, in properly designed randomized clinical trials, to reach the highest level of clinical evidence.

## Figures and Tables

**Table 1:** Common species of the genus *Trillium*.

No.	Species with Common Name	Occurrence	Flowering Period
1	*Trillium erectum* L. Wake robin Red *Trillium*	North America	April–June
2	*Trillium nivale* Riddell Snow *Trillium* Dwarf white *Trillium*	United States	March–April
3	*Trillium undulatum* Willd. Painted *Trillium* Painted lady	Wisconsin (U.S.)	April–June
4	*Trillium pusillum* Michx. Dwarf *Trillium* Least *Trillium*	United States	March–May
5	*Trillium grandiflorum* (Michx.) Salisb. Great white *Trillium* White wake-robin	Mountains of Virginia (U.S.)	April–June
6	*Trillium ovatum* Pursh Western white *Trillium*	North America	March–May
7	*Trillium luteum* Harb. Yellow *Trillium* Yellow toadshade	Joseph rivers and elsewhere in Michigan (U.S.)	April–May
8	*Trillium petiolatum* Pursh Purple *Trillium* Round-leaved *Trillium*	North America	April–May
9	*Trillium simile* Gleason Sweet white *Trillium*	North America	April–May
10	*Trillium lancifolium* Raf. Lance leaved *Trillium*	North America	February–May
11	*Trillium* *kamtschaticum* Pall. Ex Miyabe	Korea, Japan, Russia, North America, China	April–June
12	*Trillium tschonoskii* Maxim.	Bhutan, Japan, Korea, China	July–August
13	*Trillium taiwanense* S.S.Ying	Taiwan, China	May–June
14	*Trillium parviflorum* V.G.Sokup Small flowered *Trillium*	North America	March–May
15	*Trillium govanianum* Wall.	Bhutan, India, Nepal, China, Pakistan	April–August

**Table 2:** List of phytochemicals isolated from genus *Trillium*.

Source	Chemical Name	Chemical Structure	References
*T. govanianum*	Spirost-5-en-3-ol(diosgenin) (compound **1**)	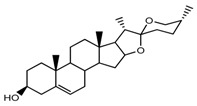	[28]
*T. erectum*	(25*S*)-spirost-5-ene-3β,17α,27-triol (compound **2**)	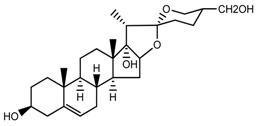	[26]
*T. erectum*	(25*S*)-3β,17α-dihydroxyspirost-5-en-27-yl β-d-glucopyranoside (compound **3**)	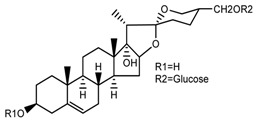	[26]
*T. erectum*	(25*S*)-17α,27-dihydroxyspirost-5-en-3 β-yl β-d-glucopyranoside (compound **4**)	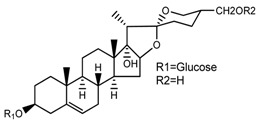	[26]
*T. erectum*	(25*S*)-27-[(β-d-glucopyranosyl)oxy]-17α-hydroxyspirost-5-en-3β-yl *O* α-l-rhamnopyranosyl-(1→2)-β-d-glucopyranoside (compound **5**)	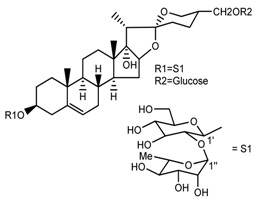	[26]
*T. erectum*	(25*S*)-27-[(β-d-glucopyranosyl)oxy]-17α,27-dihydroxyspirost-5-en-3-yl *O*-(4-*O*-acetyl-α-l-rhamnopyranosyl)-(1→2)-β-d-glucopyranoside (compound **6**)	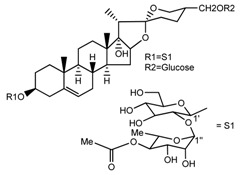	[26]
*T. erectum*	(25*S*)-27-[(β-d-glucopyranosyl)oxy]-17α,27-dihydroxyspirost-5-en-3β-d-glucopyranosyl-(1→6)-*O*-[α-l-rhamnopyranosyl-(1→2)]-β-d-glucopyranoside (compound **7**)	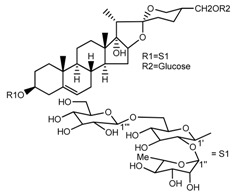	[26]
*T. erectum*	(25*S*)-17α,27-dihydroxyspirost-5-en-3β-yl *O*-(4-*O*-acetyl-α-l-rhamnopyranosyl)-(1→2)-β-d-glucopyranoside (compound **8**)	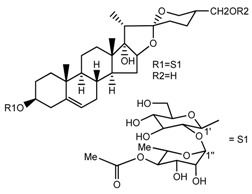	[26]
*T. erectum*	(25*R*)-17α-hydroxyspirost-5-en-3β-yl *O*-α-l-rhamnopyranosyl-(1→4)-*O*-[α-l-rhamnopyranosyl-(1→4)]-β-d-glucopyranoside (compound **9**)	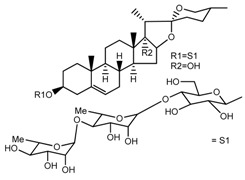	[26]
*T. erectum*	(25*R*)-26-[(β-d-glucopyranosyl)oxy]-22α-methoxyfurost-5-en-3β-yl *O*-α-l-rhamnopyranosyl-(1→2)-*O*-[α-l-rhamnopyranosyl-(1→4)]-β-d-glucopyranoside (compound **10**)	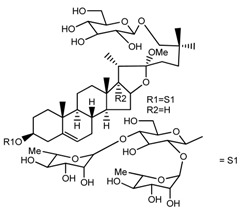	[26]
*T. kamtschaticum*	(25*S*)-17α,27-dihydroxyspirost-5-en-3β-yl *O*-α-l-rhamnopyranosyl-(1→2)-β-d-glucopyranoside (compound **11**)	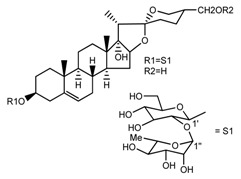	[35]
*Trillium kamtschaticum*	(25*R*)-17α-hydroxyspirost-5-en-3 β-yl *O*-α-l-rhamnopyranosyl-(1→2)-β-d-glucopyranoside (compound **12**)	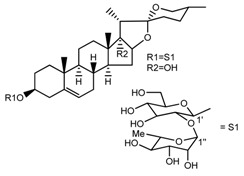	[36]
*T. kamtschaticum*	(25*R*)-17α-hydroxyspirost-5-en-3β-yl *O*-α-l-rhamnopyranosyl-(1→4)-β-d-glucopyranoside (compound **13**)	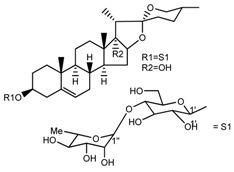	[36]
*T. kamtschaticum*	(25*R*)-17α-hydroxyspirost-5-en-3β-yl *O*-α-l-rhamnopyranosyl-(1→2)-*O*-[α-l-rhamnopyranosyl-(1→4)]-β-d-glucopyranoside (compound **14**)	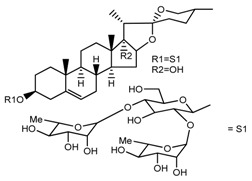	[36]
*T. kamtschaticum*	(25*R*)-17α-hydroxyspirost-5-en-3β-yl *O*-α-l-rhamnopyranosyl-(1→2)-*O*-[*O*-α-l-rhamnopyranosyl-(1→4)-a-l-rhamnopyranosyl-(1→4)]-α-d-glucopyranoside (compound **15**)	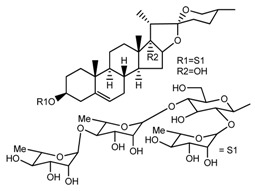	[36]
*T. kamtschaticum*	(25*R*)-spirost-5-en-3β-yl *O*-α-l-rhamnopyranosyl-(1→2)-β-d-glucopyranoside (compound **16**)	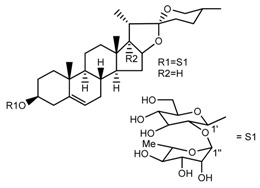	[36]
*T. kamtschaticum*	(25*R*)-spirost-5-en-3β-yl *O*-α-l-rhamnopyranosyl-(1→2)-*O*-[α-l-rhamnopyranosyl-(1→4)]-β-d-glucopyranoside (compound **17**)	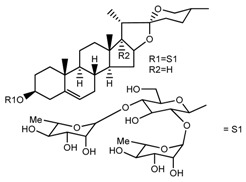	[36]
*T. kamtschaticum*	(25*R*)-spirost-5-en-3β-yl *O*-α-l-rhamnopyranosyl-(1→2)-*O*-[*O*-α-l-rhamnopyranosyl-(1→4)-α-Lrhamnopyranosyl-(1→4)]-β-d-glucopyranoside (compound **18**)	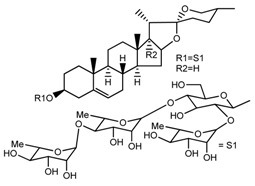	[36]
*T. kamtschaticum* *T. erectum*	(25*R*)-26-[(β-d-glucopyranosyl)oxy]-17α-hydroxy-22β-methoxyfurost-5-en-3β-yl *O*-α-l-rhamnopyranosyl-(1→2)-β-d-glucopyranoside (compound **19**)	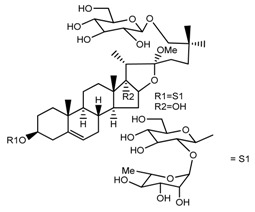	[26,37]
*T. kamtschaticum* *T. erectum*	(25*R*)-26-[(β-d-glucopyranosyl)oxy]-17α-hydroxy-22-amethoxyfurost-5-en-3β-yl *O*-α-l-rhamnopyranosyl-(1→2)-*O*-[α-l-rhamnopyranosyl-(1→4)]-β-d-glucopyranoside (compound **20**)	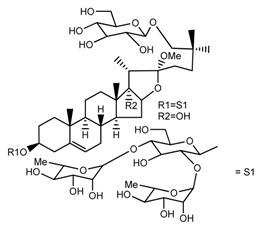	[26,37]
*T kamtschaticum*	(25*R*)-26-[(β-d-glucopyranosyl)oxy]-3β-[(*O*-α-l-rhamnopyranosyl-(1→2)-β-d-glucopyranosyl)oxy]-cholesta-5,17-diene-16,22-dione (compound **21**)	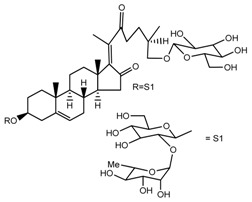	[36]
*T. kamtschaticum*	(25*R*)-27-hydroxypenogenin 3-*O*-α-l-rhamnopyranosyl-(1→2)-*O*-β-d-glucopyranoside (compound **22**)	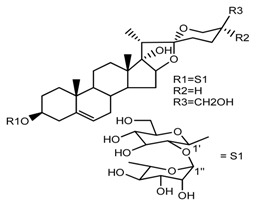	[35]
*T. kamtschaticum*	Penogenin 3-*O*-α-l-rhamnopyranosyl-(1→2)-*O*-β-d-glucopyranoside (compound **23**)	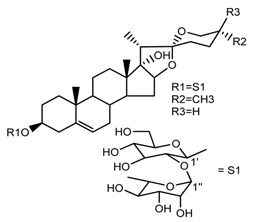	[36]
*T. kamtschaticum*	Penogenin 3-*O*-β-d-glucopyranosyl-(1→6)-[*O*-α-l[-rhamnopyranosyl-(1→2)]-*O*-β-d-glucopyranoside] (compound **24**)	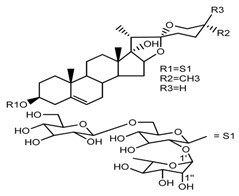	[35]
*T. kamtschaticum*	Penogenin 3-[*O*-β-[d-glucopyranoside] (compound **25**)	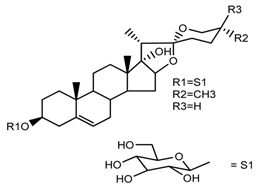	[36]
*T. kamtschaticum*	Deoxytrillenoside (compound **26**)	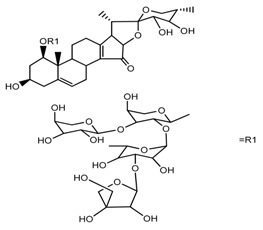	[35,38]
*T. kamtschaticum*	26-*O*-β-d-glucopyranosyl (22,25*R*)-furost-5-eene-3β,17α,22,26-tetraol 3-*O*-α-l-rhamnopyranosyl-(1→2)-*O*-β-d-glucopyranoside (compound **27**)	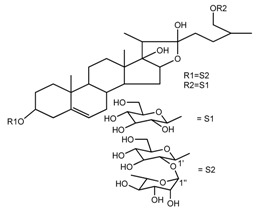	[36]
*T. kamtschaticum*	26-*O*-β-ad-23glucopyranosyl (22,25*R*)-furost-5-eene-3β,17α, 22,26-tetraol 3-*O*-α-l-rhamnopyranosyl-(1→42)-*O*-α-l-[rhamnopyranosyl-(1→04)]-*O*-β-d-glucopyranoside (compound **28**)	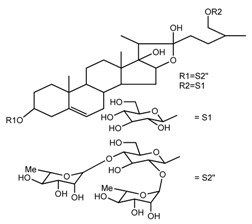	[35]
*T. kamtschaticum*	26-*O*-β-d-glucopyranosyl 17(20)-dehydrokryptogenin 3-*O*-α-l-rhamnopyranosyl-(1→2)-[*O*-α-l-rhamnopyranosyl-(1→4)]-*O*-β-d-glucopyranoside (compound **29**)	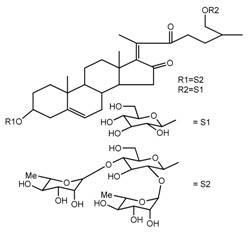	[35]
*T. kamtschaticum*	26-*O*-β-d-glucopyranosyl 17(20)-dehydrokryptogenin 3-*O*-α-l-rhamnopyranosyl-(1→2)-*O*-β-d-glucopyranoside (compound **30**)	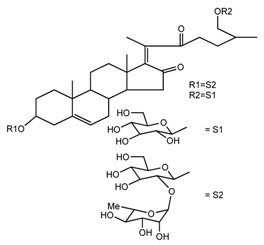	[36]
*T. govanianum*	Govanoside A (compound **31**)	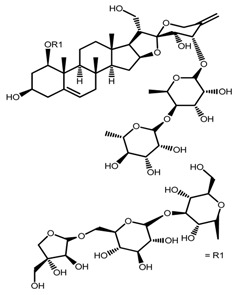	[28]
*T. govanianum*	Borassoside E (compound **32**)	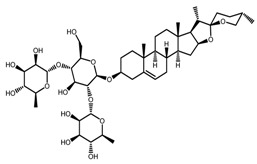	[28]
*T. tschonoskii*	l-*O*-[2,3,4-tri-*O*-acetyl-α-l-rhamnopyranosyl-(1→2)4-*O*-acetyl-α-l-arabinopyranosyl]-21-*O*-acetyl-epitrillenogenin (compound **33**)	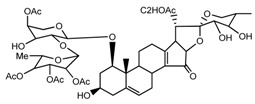	[39]
*T. tschonoskii*	(25*S*)-27-hydroxypenogenin-[3-*O*-α-l-rhamnopyranosyl-(1→2)-*O*-β-d-glucopyranoside] (compound **34**)	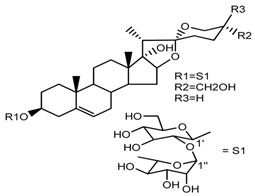	[39]
*T. govanianum* *T. grandiflorum*	Spirost-5-ene-3,17-diol(pennogenin) (compound **35**)	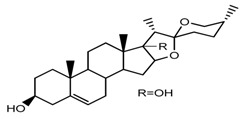	[28,40]
*T. govanianum* *T. kamtschaticum*	β-Ecdysone(20-hydroxyecdysone) (compound **36**)	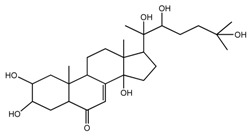	[29,35]
*T. govanianum*	5 hydroxy, β-ecdysone(5,20 dihydroxyecdysone) (compound **37**)	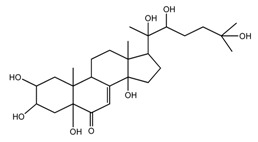	[19]
**Other non-steroidal compounds**			
*T. kamtschaticum*	(10*R*,6*E*)-7,11-dimethyl-3-mehyl3ene-6-dodecaene-1,2,10,11-tetraol 10-*O*-β-d-glucopyranoside (compound **38**)	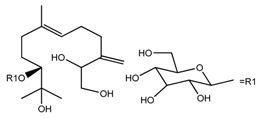	[35]
*T. kamtschaticum*	(10*R*,6*E*)-3,7,11-trimethyl-1,6-dodecadien-3,10,11-triol 10-*O*-glucopyranoside (compound **39**)	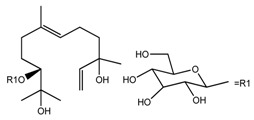	[35]
*T. kamtschaticum*	(10*R*,6*E*)-3,7,11-trimethyl-1,6-dodecadien-3,10,11-triol 10-*O*-glucopyranoside (compound **40**)	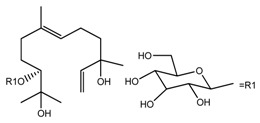	[35]
*T. tschonoskii*	7,11-dimethyl-3-methylene-1,6-dodecadien-10,11-diol 10-*O*-β-d-(1→4)glucopyranosyl-*O*-β-d-glucopyranoside (compound **41**)	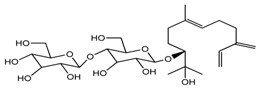	[41]
*T. tschonoskii*	Methylferulorate (compound **42**)	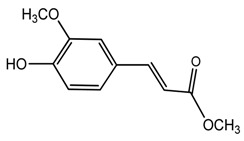	[41]
*T. kamtschaticum*	Astragalin (compound **43**)	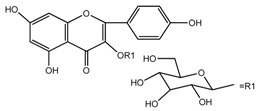	[35]
*T. undulatum*	3,4,5,7-tetrahydroxyflavone (compound **44**)	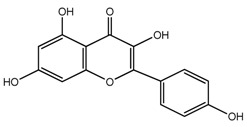	[27]
*T. undulatum*	quercetin 3-*O*-rutinoside; [3-*O*-β-l-rhamnopyranosyl-(1→6)-β-d-glucopyranoside] (compound **45**)	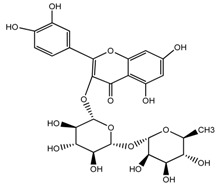	[27]
*T. undulatum*	Kaempferol 3-*O*-α-rhamnosyl-(1→2)-*O*-[α-rhamnosyl-(l→6)]-β-glucoside (compound **46**)	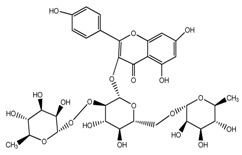	[27]
*T. tschonoskii*	*p*-hydroxymethyl benzyl alcohol (compound **47**)	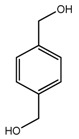	[42]
*T. govanianum*	Govanic acid (compound **48**)	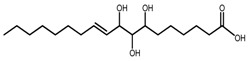	[19]
*T. tschonoskii*	3,7,11-trimethyl-3,9,11-trihydroxyl-1,6-dodecadiene glycerol (compound **49**)	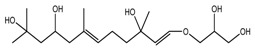	[42]
*T. tschonoskii*	2-methyl-3,4 dihydroxy-hexanedioic acid (compound **50**)	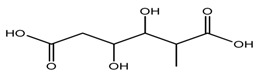	[42]

**Table 3:** Bioactivities of the genus *Trillium* phytochemicals.

Source	Compound	Reported Pharmacological Activity
*T. tschonoskii*	pennogenin 3-*O*-α-l-rhamnopyranosyl-(1→2) [α-l-rhamnopyranosyl-(1→4)]-β-d-glucopyranoside (compound **51**)	Cytotoxic, anti-proliferative and morphological effects on lung cancer cell line [52]; cytotoxicity against malignant sarcoma cells [53]
	7-β-hydroxy trillenogenin 1-*O*-β-d-apiofuranosyl-(1→3)-α-l-rhamnopyranosyl-(1→2)-[β-d-xylopyranosyl-(1→3)]-α-l-arabinopyranoside, Trillenoside A (compound **52**)	Inhibitory activity against COX-2 in in macrophagocytes of the mouse abdominal cavity stimulated by LPS [21]
*T. erectum*	(25*R*)-17α-hydroxyspirost-5-en-3β-yl *O*-α-l-rhamnopyranosyl-(1→2)-β-d-glucopyranoside (compound **53**)	Cytotoxicity against HL-60 human promyelocytic leukemia cells. IC_50_ (μg/mL) = 6.10 ± 0.04 [26]
	(25*R*)-17α-hydroxyspirost-5-en-3β-yl *O*-α-l-rhamnopyranosyl-(1→2)-*O*-[α-l-rhamnopyranosyl-(1→4)]-β-d-glucopyranoside (compound **9**)	Cytotoxicity against HL-60 human promyelocytic leukemia cells, IC_50_ (μg/mL) = 3.58 ± 0.18 [26]
	(25*R*)-17α-hydroxyspirost-5-en-3β-yl *O*-α-l-rhamnopyranosyl-(1→2)-*O*-[*O*-α-l-rhamnopyranosyl-(1→4)-α-l-rhamnopyranosyl-(1→4)]-α-d-glucopyranoside (compound **54**)	Cytotoxicity against HL-60 human promyelocytic leukemia cells, IC_50_ (μg/mL) = 2.65 ± 0.22 [26]
	(25*R*)-spirost-5-en-3β-yl *O*-α–l-rhamnopyranosyl-(1→2)-*O*-[*O*-α-l-rhamnopyranosyl-(1→4)-α–l-rhamnopyranosyl-(1→4)]-β-d-glucopyranoside (compound **55**)	Cytotoxicity against HL-60 human promyelocytic leukemia cells, IC_50_ (μg/mL) = 1.68 ± 0.11 [26]
	(25*R*)-26-[(β-d-glucopyranosyl)oxy]-22α-methoxyfurost-5-en-3β-yl *O*-α-l-rhamnopyranosyl-(1→2)-*O*-[α-l-rhamnopyranosyl-(1→4)]-β-d-glucopyranoside (methylprotodioscin) (compound **56**)	Cytotoxicity against HL-60 human promyelocytic leukemia cells, IC_50_ (μg/mL) = 2.89 ± 0.24 [26]
*T. govanianum*	(1β,3β,23*S*,24*S*)-1-[*O*-β–d-glucopyranosyl (1→3)-*O*-β-d-glucopyranosyl (1→6)-*O*-β-d-apiofuranosyl]-3,23 dihydroxyspirosta-5,25-dienyl-24-[*O*-α-l-rhamnopyranosyl (1→4)-β-d-6-deoxygulopyranoside] (govanoside A) (compound **31**) boeassoside E (compound **32**) 7, 8, 9-trihydroxy-(10*Z*)-10-octadecenoic acid (compound **48**)	Antifungal activity against *Aspergillus niger*, *A. flavus*, *Candida albicans*, *C. glabrata, Trichophyton rubrum* [19,28]
	pennogenin (compound **35**), borassoside E (compound **32**), diosgenin (compound **1**)	ROS inhibitory activity [51]
*T. kamtschaticum*	21-*O*-acetyl-trillenogenin-1-*O*-β-d-apiofuranosyl-(1→3)-4′′-acetyl-α-l-rhamnopyranosyl-(1→2)-α-l-arabinopyranoside (compound **56**)	Cytotoxicity against human colorectal cancer cells (HCT116) IC_50_ (μM) = 4.92 ± 1.00 [34]
	24-*O*-acetyl-epitrillengenin-1-*O*-β-d-apiofuranosyl-(1→3)-α-Lrhamnopyranosyl-(1→2)-[β-d-xylopyranosyl-(1→3)]-α-l-arabinopyranoside (compound **57**)	Cytotoxicity against human colorectal cancer cells (HCT116) IC_50_ (μM) = 5.84 ± 1.05 [34]
	26-*O*-β-d-glucopyranosyl-17(20)-dehydrokryptogenin-3-*O*-α-l-rhamnopyranosyl-(1→4)-β-d-glucopyranoside (compound **58**)	Cytotoxicity against human colorectal cancer cells (HCT116) IC_50_ (μM = 17.28 ± 2.69 [34]
*T. grandiflorum*	(3β,25*R*)-spirost-5-en-3-yl *O*-6-deoxy-α-l-mannopyranosyl-(1→2)-*O*-[6-deoxy-α-l-mannopyranosyl-(1→4)]-β-d-glucopyranoside (compound **59**)	Antifungal activity against *Candida albicans* MIC (μg/mL) = 1.56 [40]
